# Comparative Effectiveness of Mebeverine and Otilonium Bromide on Health-Related Quality of Life in Patients with Irritable Bowel Syndrome: A Prospective Cohort Study

**DOI:** 10.3390/life16091390

**Published:** 2026-08-24

**Authors:** Anh Thi Phuong Luu, Thong Duy Vo

**Affiliations:** 1 Department of Pharmacology, School of Pharmacy, University of Medicine and Pharmacy at Ho Chi Minh City, Ho Chi Minh City 72714, Vietnam; 2 Department of Internal Medicine, School of Medicine, University of Medicine and Pharmacy at Ho Chi Minh City, Ho Chi Minh City 72714, Vietnam; 3 Department of Gastroenterology, University Medical Center Ho Chi Minh City, Ho Chi Minh City 72714, Vietnam

**Keywords:** irritable bowel syndrome, mebeverine, otilonium bromide, quality of life, efficacy, safety

## Abstract

Irritable bowel syndrome (IBS) is a common chronic gastrointestinal disorder that significantly impairs patients’ quality of life. Antispasmodics such as mebeverine and otilonium bromide (OB) are commonly used as first-line therapies; however, comparative evidence regarding their effectiveness and safety profile remains limited. This study aimed to compare short-term changes in health-related quality of life and the occurrence of adverse events among IBS patients receiving mebeverine or otilonium bromide. A prospective cohort study was conducted from December 2024 to July 2025 among 110 IBS patients meeting the Rome IV criteria who received either mebeverine 200 mg or OB 40 mg twice daily for 4 weeks. The IBS-specific quality-of-life questionnaire (IBS-QOL) was interviewer-administered at baseline and after treatment (week 4). Adverse drug events were documented and classified according to the Common Terminology Criteria for Adverse Events (CTCAE). After 4 weeks, significant within-group improvements in the overall IBS-QOL score and most domain scores were observed in both treatment groups. In the unadjusted between-group analysis, the mean improvement in the overall IBS-QOL score was greater in the mebeverine group than in the otilonium bromide group (20.31 ± 13.17 vs. 14.62 ± 14.61; *p* = 0.034). However, treatment group was not independently associated with change in the overall IBS-QOL score after multivariable adjustment (OB vs. mebeverine: B = −4.63, 95% CI −10.28 to 1.02; *p* = 0.107). Nominal differences in activity interference and health worry did not remain statistically significant after Holm correction for multiple domain-level comparisons. Adverse events occurred in 9 of 110 patients (8.2%), with no significant difference between the mebeverine and otilonium bromide groups (11.7% vs. 4.0%; Fisher’s exact *p* = 0.178). All reported events were mild. In this prospective observational cohort, both mebeverine and otilonium bromide use was associated with short-term improvement in health-related quality of life and low rates of mild adverse events. Although the unadjusted change in the overall IBS-QOL score favored mebeverine, the adjusted analysis did not establish an independent difference between the two treatments.

## 1. Introduction

Irritable bowel syndrome (IBS) is a chronic functional gastrointestinal disorder characterized by recurrent abdominal pain associated with altered bowel movements in the absence of structural abnormalities. Common symptoms include abdominal pain, bloating, changes in stool frequency or consistency such as diarrhea, constipation, or alternating patterns of both [1]. The diagnosis of IBS is symptom-based and follows the Rome IV criteria, which require recurrent abdominal pain associated with defecation or changes in stool frequency or consistency for at least three months. IBS is further classified into four subtypes according to predominant bowel patterns: constipation-predominant (IBS-C), diarrhea-predominant (IBS-D), mixed type (IBS-M), and unclassified (IBS-U) [2]. The pathophysiology of IBS is complex and multifactorial, involving disturbances in the gut–brain axis, altered immune responses, gut microbiome dysbiosis, and psychological factors such as anxiety and depression [3,4,5,6].

Given the multifactorial nature, IBS affects a substantial proportion of the population worldwide. The prevalence of IBS varies across regions, with the highest rates reported in Southern Europe, South America, and Africa (15.0–21.0%), followed by 11.8–14.0% in North America and Northern Europe, and approximately 7% in Southeast Asia [7]. In Vietnam, recent studies reported IBS prevalence ranging from 5.5–14.4%, depending on the diagnostic criteria and populations examined, suggesting that IBS represents a significant public health issue within the country [8].

Despite being non-fatal, IBS imposes a substantial burden on patients’ quality of life due to its chronic and relapsing nature. Studies have reported that symptoms may persist for 1–2 years [9,10] and can last up to 10 years in 50–70% of the patients [11]. Persistent abdominal discomfort, psychological distress, and disruption of daily activities collectively impair patients’ well-being [12]. Consequently, improving quality of life, as assessed by the validated IBS-specific Quality of Life (IBS-QOL) questionnaire, has become a key therapeutic objective [13]. Current clinical guidelines recommend a stepwise management approach, emphasizing patient education, dietary modification, and pharmacologic therapy tailored to predominant symptoms [14,15].

Antispasmodics remain central to managing abdominal pain and discomfort. Common agents include mebeverine, otilonium bromide, pinaverium, alverine, and hyoscine. Their use varies by region—Asian countries, including Vietnam, tend to prescribe a wider range, with mebeverine and otilonium bromide being the most common [14,15,16].

Mebeverine, a β-phenylethylamine derivative used for IBS since 1965, acts mainly by reducing calcium influx in smooth muscle cells, modulating sodium channels, and exerting mild local anesthetic effects [17,18]. Clinical studies show mixed results—some report symptom relief and quality-of-life improvement comparable to agents like ramosetron and trimebutine [17,18,19,20], while others find no benefit over placebo [21,22]. In terms of safety, a meta-analysis of mebeverine reported no serious adverse effects as mebeverine acts locally on the gastrointestinal tract, which reduces the risk of systemic adverse reactions [22]. Typical doses of mebeverine for IBS are 200 mg twice daily or 135 mg three times daily, with similar efficacy between the two regimens [23].

Otilonium bromide (OB), on the other hand, primarily accumulates in the inner circular muscle layer and the submucosal layer of the colon. Its complex mechanism of action involves inhibition of calcium influx into smooth muscle cells and antagonism of muscarinic receptors and tachykinin NK_2_ receptors and may contribute to reducing intestinal smooth muscle spasms and secretion from epithelial cells [24,25,26,27]. Clinical studies have shown that OB significantly reduces abdominal pain, bloating, and overall IBS symptoms compared to placebo [28]. Adverse effects of OB, mainly due to peripheral anticholinergic activity, include dry mouth, nausea, and dizziness [25]. The typical dose is 40 mg two to three times daily [29].

Mebeverine and OB are well-established antispasmodics with proven benefits in relieving IBS symptoms and improving patient quality of life. However, direct comparative data on their efficacy and safety remain limited. In Vietnam, evidence regarding the effectiveness, particularly regarding patients’ quality of life, is lacking. Therefore, this study aimed to directly compare the efficacy and safety of mebeverine and OB in improving the quality of life of Vietnamese patients with IBS.

## 2. Material and Methods

### 2.1. Study Design

A prospective cohort study was conducted at the University Medical Center, Ho Chi Minh City, Vietnam, from December 2024 to July 2025. Adults (≥18 years) diagnosed with IBS of any subtype according to Rome IV criteria who were prescribed mebeverine or OB and provided informed consent were included. Treatment selection was determined by attending physicians according to routine clinical practice, and patients were categorized by the prescribed medication. All participants were fully informed about the study objectives and procedures before signing the informed consent and being enrolled. Exclusion criteria included organic gastrointestinal diseases, major systemic or psychiatric disorders, pregnancy or lactation, and concomitant use of drugs affecting intestinal motility, or prior use of mebeverine/OB within four weeks. The study was approved by the Institutional Review Board of University of Medicine and Pharmacy at Ho Chi Minh City (Code: 2757/ĐHYD-HDDD).

### 2.2. Sample Size

The required sample size for each group was calculated using the following formula:

$$n\;=\;2\;\times \;{\left(\frac{\left(Z2\alpha \;+\;Z2\beta \right)\;\times \;\sigma \;}{\delta }\right)}^{2}$$

- $Z_{2\alpha }\;=\;1.96,$ corresponding to a significance level of α = 0.05 (95% confidence level).
- $Z_{2\beta }\;=\;1.04$, corresponding to β = 0.2 (statistical power of 80%).
- σ = 15.3, the standard deviation of the IBS-QOL score according to the study by Nguyen et al. [5].
- δ = 10, the minimum clinically meaningful difference expected between the two groups.

### 2.3. Data Collection and Measurements

Data were collected at baseline and after 4 weeks of treatment. Baseline assessment included demographic characteristics (sex, age, occupation, marital status, place of residence), lifestyle factors (stress, smoking, consumption of coffee, alcohol, or carbonated beverage, level of physical activity), and IBS-related characteristics (subtype and concurrent therapies) using interviewer-administered questionnaires. Residences were classified as urban (city/town) or rural (commune/village). Stress and smoking status were self-reported as yes/no. Alcohol, coffee, carbonated beverage consumption, and physical activity were categorized as frequent, occasional, or none.

IBS patients’ quality of life was assessed at baseline using an interviewer-administered IBS-specific questionnaire (IBS-QOL) [13]. The instrument contains 34 items that assess overall QOL and eight domains: dysphoria, interference with activity, body image, health worry, food avoidance, social reaction, sexual concerns, and relationships. Each item is rated on a five-point Likert scale from 1 (“not at all”) to 5 (“extremely”), reflecting the impairment experienced by the patient. Raw scores for the total scale and each domain were converted to a 0–100 scale using the following formula, with higher scores indicating better quality of life.

Raw score conversion:

$$\mathrm{Converted}\;\mathrm{score}\;=\;\frac{\mathrm{Maximum}\;\mathrm{possible}\;\mathrm{total}\;\mathrm{score}-\mathrm{Actual}\;\mathrm{total}\;\mathrm{score}}{\mathrm{Score}\;\mathrm{range}}\;\times \;100$$

- Maximum possible total score = number of items × highest score (5 points).
- Score range = (Highest score − Lowest score) × number of items.

Quality of life was classified according to the 0–100 scale as follows: <50 (Very poor), 50–70 (Poor), 70–90 (Moderate), and 90–100 (Good). After 4 weeks, patients’ quality of life was reassessed using the same questionnaire via face-to-face interview or by telephone. The change in IBS-QOL score (ΔIBS-QOL) was calculated for each domain and the total score to evaluate improvement after treatment.

Adverse drug events (ADE) were recorded throughout the four-week treatment period based on patient self-reporting. All reported events were classified by study investigators as mild, moderate or severe based on the Common Terminology Criteria for Adverse Events (CTCAE), version 5.0, to determine their type and severity [30].

### 2.4. Statistical Analysis

Statistical analyses were performed using SPSS Statistics version 27.0. Continuous variables were expressed as mean ± standard deviation or median with interquartile range, depending on their distribution, and categorical variables were presented as frequencies and percentages. Within-group changes in IBS-QOL scores were assessed using paired *t*-tests or Wilcoxon signed-rank tests, as appropriate. Between-group differences were assessed using independent-samples *t*-tests or Mann–Whitney U tests.

Change in the overall IBS-QOL score was considered the principal outcome. The eight domain-level comparisons were considered secondary exploratory analyses, and their *p*-values were adjusted using the Holm procedure. Adverse-event rates were compared using the two-sided Fisher’s exact test because of the small cell counts.

Treatment group was entered into the multivariable linear regression model a priori. Age, stress level, marital status, exercise, and concomitant use of sulpiride, trimebutine, and rifaximin were included based on clinical relevance, univariable association, or imbalance between treatment groups at baseline. Given the observational design, the resulting estimates were interpreted as adjusted associations rather than causal treatment effects. All tests were two-sided, and *p* < 0.05 was considered statistically significant.

## 3. Results

A total of 110 patients were enrolled, including 60 receiving mebeverine and 50 receiving otilonium bromide (Table 1). Baseline demographic and lifestyle characteristics were generally similar between the two treatment groups. However, the distribution of exercise frequency differed significantly between groups (*p* = 0.002). Trimebutine and rifaximin were also prescribed more frequently in the mebeverine group (*p* = 0.019 and *p* = 0.002, respectively). These baseline imbalances were considered in the multivariable analysis.

Table 2 summarizes baseline and week-4 IBS-QOL total and domain scores, along with quality-of-life classifications, for patients receiving mebeverine and OB. Both groups showed substantial increases in median total IBS-QOL scores after 4 weeks, with most patients improving from ‘very bad/bad’ to ‘good’ quality-of-life categories. Similar significant within-group improvements were observed across all domains, including dysphoria, activity interference, body image, health worry, food avoidance, social reaction, sexual concerns and relationship (all *p* < 0.001 for mebeverine, and *p* ≤ 0.006 for OB).

Table 3 compares the changes in IBS-QOL scores by domain after 4 weeks of treatment. The mean improvement in total IBS-QOL score was greater in the mebeverine group than in the OB group (20.31 ± 13.17 vs. 14.62 ± 14.61; *p* = 0.034). Domain analysis showed significantly larger improvement with mebeverine in activity interference and health worry (*p* = 0.043 and *p* = 0.029, respectively), whereas changes in dysphoria, body image, food avoidance, social reaction, sexual concern, and relationship domains did not differ significantly between the two groups (all *p* > 0.05).

Adverse events were reported in 9 of 110 patients (8.2%), including 7 of 60 patients receiving mebeverine and 2 of 50 patients receiving otilonium bromide (Table 4). The difference between groups was not statistically significant (11.7% vs. 4.0%; two-sided Fisher’s exact *p* = 0.178). All reported events were mild, and no treatment discontinuation due to an adverse event was recorded.

**Table 4:** Adverse events during 4 weeks of treatment.

Adverse Events	Total (*n* = 110)	Groups [n (%)]		*p*-Value
		Mebeverine (*n* = 60)	OB (*n* = 50)	
**Any adverse event** Nausea Dizziness Gastrointestinal discomfort Abdominal pain Insomnia Pruritus	9 (8.2%) 2 (1.8%) 2 (1.8%) 2 (1.8%) 1 (0.9%) 1 (0.9%) 1 (0.9%)	7 (11.7) 2 (3.3) 1 (1.7) 1 (1.7) 1 (1.7) 1 (1.7) 1 (1.7)	2 (4.0) 0 (0.0) 1 (2.0) 1 (2.0) 0 (0.0) 0 (0.0) 0 (0.0)	0.178 - - - - - -
**No adverse event**	101 (91.8%)	53 (88.3)	48 (96.0)	

The *p*-value for any adverse event was calculated using the two-sided Fisher’s exact test. Individual adverse-event types were presented descriptively because of the small event counts.

Table 5 shows the factors associated with change in overall IBS-QOL score. In the univariable analysis, receiving otilonium bromide rather than mebeverine was associated with a 5.82-point smaller improvement in the overall IBS-QOL score (B = −5.82, 95% CI −11.06 to −0.58; *p* = 0.030). Marital status and concomitant sulpiride use were also associated with change in the overall IBS-QOL score. After multivariable adjustment, treatment group was no longer statistically significant (B = −4.63, 95% CI −10.28 to 1.02; *p* = 0.107). Concomitant sulpiride use remained associated with a greater change in the overall IBS-QOL score (B = 6.72, 95% CI 0.30 to 13.14; *p* = 0.040). Given the non-random treatment allocation, the sulpiride finding should be considered exploratory and should not be interpreted as a causal treatment effect.

## 4. Discussion

In this prospective observational cohort, both treatment groups demonstrated significant within-group improvements in the overall IBS-QOL score and most quality-of-life domains after 4 weeks, with low rates of mild adverse events. Although the unadjusted change in the overall IBS-QOL score was greater in the mebeverine group, treatment group was not significantly associated with quality-of-life change after multivariable adjustment. In addition, the nominal between-group differences in activity interference and health worry did not remain statistically significant after correction for multiple comparisons.

### 4.1. Efficacy of Mebeverine on Quality of Life

In our study, mebeverine was associated with a clear and clinically meaningful improvement in health-related quality of life in patients with IBS. The average gain in overall IBS-QOL score over 4 weeks was well above the minimal important difference, and all core domains—including emotional distress, daily activities, body image, health concerns, dietary restriction, and social functioning—showed significant improvement. These findings are broadly consistent with an international prospective cohort of 607 IBS patients by Hou et al. in which mebeverine led to statistically and clinically meaningful gains in IBS-QOL total and subscale scores after 4 and 8 weeks of treatment [31]. A more recent systematic review likewise concluded that mebeverine provides effective symptom relief in IBS and has favorable effects on patient-reported outcomes, with a low frequency of adverse events [32]. Differences in the absolute magnitude of change between our study and multicenter cohorts are likely influenced by heterogeneity in study design, concomitant use of other antispasmodics, variation in doses and formulations, and differences in how quality-of-life data were collected. For example, fully self-completed electronic questionnaires may reduce interviewer bias but provide less opportunity to clarify items for anxious patients or those with limited health literacy, whereas our interviewer-assisted approach probably ensured more consistent understanding of the scale [33,34]. Trials in other populations, including younger adults with diarrhea-predominant IBS, have also demonstrated significant, though sometimes smaller, improvements in IBS-QOL with mebeverine [18]. Variation in effect size across studies likely reflect differences in age distribution, employment status, educational level, and IBS subtype, all of which shape how much IBS interferes with work, social engagement, and self-image, and therefore how strongly patients perceive change [35,36,37]. Overall, across diverse designs and populations, existing evidence, together with our data, supports that mebeverine is a well-tolerated treatment that can meaningfully enhance quality of life in patients with IBS, while highlighting the need for a larger, more standardized multicenter trials to better quantify these benefits.

### 4.2. Efficacy of Otilonium Bromide on Quality of Life

In our cohort, OB also led to substantial improvements in quality of life, with significant increases in overall IBS-QOL score and across most domains after 4 weeks of treatment. Although the number of studies specifically evaluating IBS-QOL with OB is limited, our results are broadly consistent with a randomized trial from Spain, in which prolonged treatment with OB produced mean increases in IBS-QOL scores of similar magnitude, even though differences versus placebo did not reach statistical significance [38]. Furthermore, other trials and a pooled analysis have demonstrated that OB significantly reduces abdominal pain, bloating, and altered bowel habits compared with placebo, outcomes that are closely linked to quality of life in IBS [29,39]. Taken together, these data suggest that otilonium bromide provides meaningful improvements in overall quality of life and its key domains in IBS, with an effect size comparable to that observed in international studies, while highlighting that dedicated QoL-focused trials with this agent remain relatively scarce.

### 4.3. Mebeverine Versus Otilonium Bromide

Both treatment groups demonstrated clinically meaningful within-group improvements in the overall IBS-QOL score over 4 weeks. Although the unadjusted change in the overall score was greater in the mebeverine group, treatment group was not independently associated with quality-of-life improvement after multivariable adjustment. Moreover, the nominal between-group differences in activity interference and health worry did not remain statistically significant after correction for multiple comparisons. Therefore, the present findings do not establish the superiority of mebeverine over otilonium bromide.

The attenuation of the between-group difference after adjustment may partly reflect the non-random allocation of treatment and baseline differences in exercise habits and concomitant medication use. Accordingly, the observed unadjusted difference should be considered hypothesis-generating and requires confirmation in adequately powered randomized head-to-head trials.

Our findings are broadly consistent with a previous randomized head-to-head trial comparing mebeverine and otilonium bromide. In that 8-week study of 117 Asian patients, both treatments significantly reduced abdominal pain or discomfort and improved other IBS symptoms, while otilonium bromide was non-inferior to mebeverine in relieving pain, flatulence, and bloating, with comparable safety [40]. Unlike that trial, our study primarily evaluated health-related quality of life. Nevertheless, because of its observational design and the attenuation of the treatment-group difference after adjustment, our study cannot establish that either antispasmodic provides superior quality-of-life benefits.

### 4.4. Adverse Events

Adverse events were uncommon in both treatment groups over 4 weeks. No statistically significant difference in the occurrence of adverse events was observed between the mebeverine and otilonium bromide groups (11.7% vs. 4.0%; two-sided Fisher’s exact *p* = 0.178). The few reported events—nausea, dizziness, gastrointestinal discomfort, abdominal pain, insomnia, and pruritus—were mild, non-serious, and did not lead to drug discontinuation, which is consistent with previous reports that mebeverine and otilonium bromide have favorable safety profiles [32,41]. Compared with the trial by Chang et al., we observed a higher frequency of nausea and dizziness with mebeverine but lower frequencies of abdominal pain, insomnia, pruritus, and gastrointestinal discomfort, while in the otilonium bromide group only dizziness and gastrointestinal discomfort were reported and we did not detect headache, myalgia, or dry mouth [40]. These differences may reflect the shorter follow-up (4 vs. 8 weeks) and lower daily dose of otilonium bromide in our study (40 mg twice daily versus 40 mg three times daily), which could reduce the likelihood of dose-dependent or late-onset reactions [29]. Overall, our findings support the view that both antispasmodics offer a good balance between efficacy and safety in IBS, although longer-term studies are needed to more fully characterize rare or delayed adverse events.

### 4.5. Factors Associated with QoL Improvement

In the univariable analysis, treatment group, marital status, and concomitant sulpiride use were associated with change in the overall IBS-QOL score. After multivariable adjustment, treatment group and marital status were no longer statistically significant. Concomitant sulpiride use remained associated with a greater change in the overall IBS-QOL score (B = 6.72, 95% CI 0.30 to 13.14; *p* = 0.040). However, this finding should be regarded as an exploratory association rather than evidence of an independent treatment effect because sulpiride was not randomly assigned and its indication was not systematically recorded.

Previous studies have reported improvements in IBS symptoms and quality of life among patients receiving sulpiride [42,43,44]. Sulpiride is a benzamide derivative with dopamine D2 receptor antagonist activity and may influence gastrointestinal motility and brain–gut interactions [45,46]. These observations provide biological plausibility for the association observed in our cohort but do not establish a causal treatment effect.

Sulpiride was prescribed according to the attending physician’s clinical judgment and may have been preferentially given to patients with greater psychological distress, more severe symptoms, or poorer baseline quality of life. Such patients may have had greater opportunity for improvement, and part of the observed change may therefore reflect regression to the mean. Because baseline psychological burden, symptom severity, and the clinical indication for sulpiride were not systematically measured, confounding by indication and residual confounding cannot be excluded. Further studies specifically designed to evaluate sulpiride are required to confirm this exploratory finding.

## 5. Strengths and Limitations

This study has several strengths. It used a prospective comparative design with systematic follow-up, applied a validated IBS-QOL questionnaire with domain-level analysis, and was conducted in a large tertiary referral center in Vietnam, providing real-world data for an Asian IBS population. To our knowledge, it is also the first study in Vietnam—and one of the first in Asia—to directly compare the efficacy of mebeverine and otilonium bromide in terms of health-related quality of life.

Several limitations should be acknowledged. First, treatment was not randomly assigned but was selected by attending physicians according to routine clinical practice. The observed differences in exercise habits and concomitant trimebutine and rifaximin use indicate that the treatment groups were not fully comparable at baseline. Although these variables were considered in the multivariable model, residual confounding and confounding by indication cannot be excluded. Second, sulpiride was also prescribed according to clinical judgment, and the observed association with quality-of-life improvement may partly reflect greater baseline psychological distress or symptom severity, greater opportunity for improvement, or regression to the mean. Third, the domain-level analyses involved multiple secondary comparisons, and none of the nominal between-group differences remained statistically significant after Holm correction. Finally, the single-center setting, modest sample size, and 4-week follow-up limit the generalizability of the findings and preclude conclusions regarding long-term effectiveness or uncommon adverse events.

## 6. Conclusions

In this prospective observational cohort, both mebeverine and otilonium bromide use was associated with short-term improvement in health-related quality of life and low rates of mild adverse events. Although the unadjusted change in the overall IBS-QOL score favored mebeverine, treatment group was not significantly associated with quality-of-life change after multivariable adjustment. Moreover, the nominal between-group differences in individual IBS-QOL domains did not remain statistically significant after correction for multiple comparisons. Therefore, the present findings do not establish the superiority of either antispasmodic. The exploratory association between concomitant sulpiride use and greater quality-of-life improvement requires confirmation in specifically designed prospective studies.

## Figures and Tables

**Table 1:** Baseline demographic characteristics.

Characteristics	Groups			*p*-Value
	Total (*n* = 110)	Mebeverine (*n* = 60)	Otilonium Bromide (*n* = 50)	
**Demographic characteristics**				
**Sex** Male Female	52 (47.3) 58 (52.7)	30 (50.0) 30 (50.0)	22 (44.0) 28 (56.0)	0.429
**Age**	45.5 ± 14.7	45.4 ± 15.8	45.6 ± 13.4	0.933
**Age group** Under 30 years old 30–60 years old Over 60 years old	18 (16.4) 76 (69.1) 16 (14.5)	12 (20.0) 37 (61.7) 11 (18.3)	6 (12.0) 39 (78.0) 5 (10.0)	0.181
**Place of residence** Urban area Rural area	25 (22.7) 85 (77.3)	14 (23.3) 46 (76.7)	11 (22.0) 39 (78.0)	0.868
**Occupations** Farmers Workers Businessman Office workers Teachers Students Others No current ocupation *	11 (10.0) 4 (3.6) 18 (16.4) 15 (13.6) 6 (5.5) 6 (5.5) 13 (11.8) 36 (32.7)	3 (6.7) 2 (3.3) 7 (11.7) 11 (18.3) 3 (5.0) 5 (8.3) 7 (11.7) 21 (54.5)	8 (16.0) 2 (4.0) 11 (22.0) 4 (8.0) 3 (6.0) 1 (2.0) 6 (12.0) 15 (45.5)	0.299
**Marital status** Single/Divorced Married	17 (15.5) 93 (84.5)	11 (18.3) 49 (81.7)	6 (12.0) 44 (88.0)	0.360
**Lifestyle characteristics**				
**Stress** Yes No	55 (50.0) 55 (50.0)	28 (46.7) 32 (53.3)	27 (54.0) 23 (46.0)	0.444
**Smoking** Yes No	10 (9.1) 100 (90.9)	5 (8.3) 55 (91.7)	5 (10.0) 45 (90.0)	0.762
**Alcohol use** Frequent ^a^ Occasional ^a^ None	4 (3.6) 20 (18.2) 86 (78.2)	3 (5.0) 13 (21.7) 44 (73.3)	1 (2.0) 7 (14.0) 42 (84.0)	0.377
**Carbonated drinks** Frequent ^a^ Occasional ^a^ None	7 (6.4) 22 (20.0) 81 (73.6)	4 (6.7) 15 (25.0) 41 (68.3)	3 (6.0) 7 (14.0) 40 (80.0)	0.337
**Coffee** Frequent ^a^ Occasional ^a^ None	18 (16.3) 20 (18.2) 72 (65.5)	10 (16.7) 12 (20.0) 38 (63.3)	8 (16.0) 8 (16.0) 34 (68.0)	0.844
**Exercise** Frequent ^b^ Occasional ^b^ None—total	60 (54.5) 28 (25.4) 22 (20.1)	35 (58.3) 20 (33.3) 5 (8.3)	25 (50.0) 8 (16.0) 17 (34.0)	0.002
**IBS-related characteristic**				
**IBS subtypes** IBS-C IBS-D IBS-M IBS-U	13 (11.8) 59 (53.7) 13 (11.8) 25 (22.7)	7 (11.7) 29 (48.3) 8 (13.3) 16 (26.7)	6 (12.0) 30 (60.0) 5 (10.0) 9 (18.0)	0.604
**IBS medications** Sulpiride Trimebutine Antidiarrheal ^c^ Anti-constipation ^d^ Probiotics ^e^ Rifaximin	25 (22.7) 44 (40.0) 1 (0.9) 3 (2.7) 53 (48.2) 20 (18.2)	14 (23.3) 30 (68.2) 1 (1.7) 2 (3.3) 29 (48.3) 17 (28.3)	11 (22.0) 14 (31.8) 0 (0.0) 1 (2.0) 24 (48.0) 3 (6.0)	0.868 0.019 0.359 0.669 0.972 0.002

^a^ Frequent: ≥3 times per week, occasional: <3 times per week. ^b^ Frequent: at least 30 min daily, 3–4 days per week, occasional: 2–4 days per week. ^c^ Antidiarrheal: loperamide. ^d^ Anti-constipation: PEG (polyethylene glycol) or lubiprostol. ^e^ Probiotics: *Saccharomyces boulardii* and *Bacillus claussii*. * No current occupation includes participants who were unemployed, retired, or homemakers.

**Table 2:** IBS-QOL total score, domain-specific scores, and quality-of-life classifications at baseline and after 4 weeks of treatment with mebeverine and otilonium bromide.

Domains	Mebeverine (*n* = 60)			OB (*n* = 50)		
	Baseline	Week 4	*p*-Value	Baseline	Week 4	*p*-Value
**Total** Median (Q1–Q3) <50 (Very bad), n (%) 50–70 (Bad), n (%) 70–90 (Moderate), n (%) 90–100 (Good), n (%)	74.26 (66.36–85.24) 4 (6.7) 20 (33.3) 29 (48.3) 7 (11.7)	98.16 (92.64–100.00) 0 (0.0) 3 (5.0) 9 (15.0) 48 (80.0)	<0.001	77.94 (65.99–86.76) 1 (2.0) 15 (30.0) 25 (50.0) 9 (18.0)	97.42 (85.84–100.00) 0 (0.0) 6 (12.0) 9 (18.0) 35 (70.0)	<0.001
**Dysphoria** Median (Q1–Q3) <50 (Very bad), n (%) 50–70 (Bad), n (%) 70–90 (Moderate), n (%) 90–100 (Good), n (%)	70.31 (50.00–92.97) 14 (23.3) 16 (26.7) 14 (23.3) 16 (26.7)	100.00 (93.75–100.0) 1 (1.7) 5 (8.3) 5 (4.5) 49 (81.7)	<0.001	78.12 (52.34–96.88) 10 (20.0) 11 (22.0) 16 (32.0) 13 (26.0)	100.00 (83.59–100.00) 3 (6.0) 4 (8.0) 8 (16.0) 35 (70.0)	<0.001
**Activity interference** Median (Q1–Q3) <50 (Very bad), n (%) 50–70 (Bad), n (%) 70–90 (Moderate), n (%) 90–100 (Good), n (%)	75.00 (53.57–89.29) 13 (21.7) 13 (21.7) 24 (40.0) 10 (16.7)	100.00 (83.59–100.00) 0 (0.0) 3 (5.0) 11 (18.3) 46 (76.7)	<0.001	76.79 (63.39–92.86) 5 (10.0) 15 (30.0) 17 (34.0) 13 (26.0)	100.00 (77.67–100.00) 3 (6.0) 5 (10.0) 8 (16.0) 34 (68.0)	<0.001
**Body image** Median (Q1–Q3) <50 (Very bad), n (%) 50–70 (Bad), n (%) 70–90 (Moderate), n (%) 90–100 (Good), n (%)	81.25 (75.56–93.75) 3 (5.0) 10 (16.7) 28 (46.7) 19 (31.7)	100.00 (92.86–100.00) 0 (0.0) 1 (1.7) 5 (8.3) 54 (90.0)	<0.001	81.25 (75.00–87.50) 1 (2.0) 9 (18.0) 29 (58.0) 11 (22.0)	100.00 (83.33–100.00) 0 (0.0) 0 (0.0) 9 (18.0) 41 (82.0)	<0.001
**Health worry** Median (Q1–Q3) <50 (Very bad), n (%) 50–70 (Bad), n (%) 70–90 (Moderate), n (%) 90–100 (Good), n (%)	50.00 (33.33–66.67) 23 (38.3) 25 (41.7) 6 (10.0) 6 (10.0)	100.00 (83.33–100.00) 1 (1.7) 7 (11.7) 12 (20.0) 40 (66.7)	<0.001	58.33 (39.58–77.08) 15 (30.0) 22 (44.0) 4 (8.0) 9 (18.0)	100.00 (72.92–100.00) 2 (4.0) 10 (20.0) 9 (18.0) 29 (58.0)	<0.001
**Food avoidance** Median (Q1–Q3) <50 (Very bad), n (%) 50–70 (Bad), n (%) 70–90 (Moderate), n (%) 90–100 (Good), n (%)	58.33 (39.58–75.00) 19 (31.7) 22 (36.7) 11 (18.3) 8 (13.3)	100.00 (75.00–100.00) 7 (11.6) 6 (10.0) 10 (16.7) 37 (61.7)	<0.001	66.67 (39.58–83.33) 14 (28.0) 17 (34.0) 12 (24.0) 7 (14.0)	100.00 (75.00–100.00) 5 (10.0) 1 (2.0) 12 (24.0) 32 (64.0)	<0.001
**Social reaction** Median (Q1–Q3) <50 (Very bad), n (%) 50–70 (Bad), n (%) 70–90 (Moderate), n (%) 90–100 (Good), n (%)	100.00 (87.50–100.00) 2 (3.3) 7 (11.7) 7 (11.7) 44 (73.3)	100.00 (100.00–100.00) 0 (0.0) 1 (1.7) 4 (6.7) 55 (91.7)	<0.001	100.00 (92.19–100.00) 1 (2.0) 3 (6.0) 8 (16.0) 38 (76.0)	100.00 (83.33–100.00) 1 (2.0) 2 (4.0) 2 (4.0) 45 (90.0)	0.006
**Sexual concerns** Median (Q1–Q3) <50 (Very bad), n (%) 50–70 (Bad), n (%) 70–90 (Moderate), n (%) 90–100 (Good), n (%)	100.00 (100.00–100.00) 2 (3.3) 1 (1.7) 3 (5.0) 54 (90.0)	100.00 (100.00–100.00) 1 (0.9) 0 (0.0) 1 (0.9) 58 (96.7)	0.041	100.00 (100.00–100.00) 0 (0.0) 0 (0.0) 5 (10.0) 45 (90.0)	100.00 (100.00–100.00) 0 (0.0) 0 (0.0) 1 (2.0) 49 (98.0)	0.011
**Relationship** Median (Q1–Q3) <50 (Very bad), n (%) 50–70 (Bad), n (%) 70–90 (Moderate), n (%) 90–100 (Good), n (%)	100.00 (100.00–100.00) 2 (3.3) 4 (6.7) 2 (3.3) 52 (86.7)	100.00 (100.00–100.00) 2 (1.8) 0 (0.0) 0 (0.0) 58 (96.7)	0.009	100.00 (100.00–100.00) 0 (0.0) 2 (4.0) 5 (10.0) 43 (86.0)	100.00 (100.00–100.00) 1 (2.0) 0 (0.0) 2 (4.0) 47 (94.0)	0.040

**Table 3:** Changes in IBS-QOL scores by domains after 4 weeks of treatment with mebeverine or OB.

Changes in IBS-QOL Scores	Groups		Unadjusted *p*-Value	Holm-Adjusted *p*
	Mebeverine (*n* = 60)	OB (*n* = 50)		
Δ Median	20.31 ± 13.17	14.62 ± 14.61	0.034	Not applicable—principal outcome
Δ Dysphoria	21.87 (6.25–48.43)	14.06 (0.00–37.50)	0.149	0.894
Δ Activity interference	21.42 (10.71–35.71)	17.85 (0.00–32.14)	0.043	0.301
Δ Body image	12.50 (6.25–18.75)	15.62 (0.00–25.00)	0.758	1.000
Δ Health worry	33.33 (18.75–56.25)	20.83 (0.00–43.75)	0.029	0.232
Δ Food avoidance	26.94 ± 28.59	25.00 (8.33–50.00)	0.800	1.000
Δ Social reaction	0.00 (0.00–6.25)	0.00 (0.00–6.25)	0.951	1.000
Δ Sexual concern	0.00 (0.00–0.00)	0.00 (0.00–0.00)	0.337	1.000
Δ Relationship	0.00 (0.00–0.00)	0.00 (0.00–0.00)	0.637	1.000

In the unadjusted comparison, the mean improvement in the overall IBS-QOL score was greater in the mebeverine group than in the otilonium bromide group (20.31 ± 13.17 vs. 14.62 ± 14.61; *p* = 0.034). Nominally greater improvements in activity interference and health worry were also observed in the mebeverine group. However, neither domain-level difference remained statistically significant after Holm correction (adjusted *p* = 0.301 and *p* = 0.232, respectively). No significant between-group differences were observed in the remaining domains.

**Table 5:** Factors associated with change in the overall IBS-QOL score.

Variables	Univariate Analysis		Multivariate Analysis	
	B (95% CI)	*p*-Value	B (95% CI)	*p*-Value
Antispasmodic treatment Otilonium bromide vs. mebeverine	−5.82 (−11.06, −0.58)	0.030	−4.63 (−10.28, 1.02)	0.107
Age, per year	−0.12 (−0.30, 0.06)	0.180	0.00 (−0.22, 0.22)	0.998
Sex	2.02 (−3.33, 7.38)	0.455	-	-
Place of residence	−3.05 (−9.39, 3.29)	0.342	-	-
Stress level	4.3 (−0.94, 9.60)	0.106	2.16 (−3.28, 7.60)	0.434
Smoking	−1.89 (−11.16, 7.38)	0.687	-	-
Alcohol use	−1.91 (−7.1, 3.23)	0.467	-	-
Carbonated drink consumption	−0.31 (−4.84, 4.21)	0.891	-	-
Caffeinated drink consumption	0.58 (−2.93, 4.09)	0.744	-	-
Exercise	1.18 (−2.18, 4.54)	0.488	0.42 (−2.97, 3.80)	0.807
Married vs. single/divorced	−8.41 (−15.61, −1.21)	0.023	−7.33 (−16.53, 2.09)	0.119
Sulpiride use, yes vs. no	6.36 (0.11, 12.61)	0.046	6.72 (0.30, 13.14)	0.040
Trimebutine	0.35 (−5.1, 5.79)	0.900	0.42 (−5.30, 6.13)	0.886
Antidiarrheal	14.81 (−13.14, 42.77)	0.296	-	-
Anticonstipation	−3.30 (−19.66, 13.06)	0.690	-	-
Probiotics	2.76 (−2.55, 8.07)	0.305	-	-
Rifaximin	2.21 (−4.69, 9.12)	0.526	1.08 (−6.35, 8.50)	0.774

Treatment group was entered into the model a priori. Other covariates were included based on clinical relevance, univariable association, or baseline imbalance between treatment groups.

## Data Availability

The data that support the findings of this study are available from the corresponding author upon reasonable request. Data are not publicly available due to privacy and ethical restrictions involving patient information.
